# Comparison of Sexual Violence Perpetration Rates Among Gay, Lesbian and Heterosexual Cisgender Adults

**DOI:** 10.1007/s13178-024-01030-2

**Published:** 2024-09-26

**Authors:** R. E. Anderson, D. M. Piggott, B. A. Feinstein, C. Dyar

**Affiliations:** 1 University of North Dakota, 501 N. Columbia Rd, Stop 8380, Grand Forks, ND 58202 USA; 2 Kent State University, Kent, USA; 3 University of Missouri-Kansas City, Health Sciences Building 2319, 2464 Charlotte St, Kansas City, MO 64108 USA; 4 Rosalind Franklin University of Medicine and Science, North Chicago, USA; 5 Ohio State University, Columbus, USA

**Keywords:** LGBTQ+, Perpetration, Sexual violence, Assault, Coercion, Aggression

## Abstract

**Introduction:**

LGBTQ+ individuals are more vulnerable to experiencing sexual victimization. To truly prevent sexual victimization, preventing perpetration is necessary. The goal of this study was to increase the inclusivity of sexual violence research by examining the rates of sexual violence perpetration among cisgender, monosexual (e.g., attracted to a single gender, heterosexual or gay/lesbian) adults.

**Methods:**

Participants were 828 adult Amazon Mechanical Turk workers who completed the study between March and September 2018. Participants completed the short version of the Post-Refusal Sexual Persistence Scale – Perpetration as part of a larger experiment. Analyses compared four gender by sexual identity groups: heterosexual women (31.5%), heterosexual men (27.8%), lesbian women (21.1%), and gay men (19.6%).

**Results:**

There were group differences in reported perpetration rates. Perpetration rates were higher in both groups of men (heterosexual = 33.0%, gay = 35.0%) compared to both groups of women (heterosexual = 12.6%, lesbian = 20.5%), but rates did not differ within gender. Among the subsample with perpetration histories, heterosexual men were more likely to report using verbal coercion tactics (52.0%) than the other groups (45.5% [heterosexual women], 31.6% [gay men], 30.6% [lesbian women]), and heterosexual women were more likely to report using both verbal coercion and physical force in combination (24.2%) than gay men (5.3%).

**Conclusions and Implications:**

There are gender-driven differences in the rate of sexual violence perpetration among monosexual adults, suggesting the need for population-specific prevention efforts.

Contact sexual violence—the experience of non-consensual sexual contact—is a mechanism underlying health disparities, as it does not affect all individuals equally (Black et al., 2011). Individuals who experience marginalization are more likely to be victimized, including women, members of the LGBTQ+ community, and many communities of color (Black et al., 2011; Chen et al., 2020; Rosay, 2016). Understanding how to prevent sexual violence has enormous potential to improve public health given that experiencing sexual victimization diminishes health in many examined domains (Dworkin et al., 2017; Koss, 1993). However, sexual violence research tends to focus on understanding victimization rather than perpetration (Abbey, 2005). Yet, a key factor in the prevention of sexual violence victimization and resulting health disparities is understanding and developing interventions that address *perpetration*. A holistic view of sexual violence is critical, including examining both victimization and perpetration and how intersecting social identities relate to risks. Thus, the goal of this study is to examine the prevalence rates for sexual violence perpetration among a sample of cisgender men and women who label their sexual orientation as monosexual (e.g., who describe their sexual orientation as attracted to one gender, e.g., heterosexual people, gay men, lesbian women).

## Prior Research on Gender, Sexual Orientation, and Perpetration Prevalence Rates

To understand who is harming LGBTQ+ individuals, data on perpetration behavior from individuals with a wide range of genders and sexual identities is needed. The majority of research on sexual perpetration comes from samples of heterosexual, cisgender college men, and approximately 30% of college men report sexual violence perpetration (Anderson et al., 2021a, 2021b, 2021c, 2021d). However, as this systematic review notes, sexual and gender minority men were routinely excluded from most of this work (Anderson, Kuhn, et al., 2021b). This may be in part due to harmful and inaccurate stereotypes that LGBTQ+ individuals are sexual predators (Mishel, 2020). Thus, researchers have likely avoided this area of research for fear of harming and to avoid managing the additional layers of scrutiny and discomfort associated with this topic. Although this avoidance may be well-intentioned, people of all genders and sexual identities can and do perpetrate sexual violence, and collecting data on this behavior among LGBTQ+ individuals is critical to efforts to prevent sexual violence. Methodologically, this research can also be difficult to conduct. Measures and procedures must be designed very carefully to allow participants to disclose these stigmatized and potentially illegal behaviors and how sexual orientation is measured (i.e., by self-label of identity, by behavior, or by attraction) must be carefully considered and described to accurately reflect participants’ realities in ways that are affirming.

Our paper seeks to begin addressing this gap in the literature. LGBTQ+ people are overrepresented as victims of sexual violence, and research on other forms of violence (e.g., intimate partner violence) demonstrates that LGBTQ+ individuals are represented on both sides of the victim/perpetrator dyad (Chen et al., 2020; Stotzer, 2009). We purposefully use the term LGBTQ+ in describing violence involvement to demonstrate the many different sexual minority subpopulations that have been identified in prior research as being sexually victimized at higher rates than the general population, suggesting perpetrators target them (Wiginton et al., 2023). However, we wish to clarify that at other points in the paper, we may use umbrella terms like “sexual minority” to describe studies that collapsed identity groups or measured sexual orientation in other ways, such as by sexual behavior.

As well characterized in the intimate partner violence literature, violence involvement is often dualistic, in other words, being involved with *both* victimization and perpetration because of shared vulnerability factors such as substance use, attitudes towards violence, and violence-supportive policies (Moylan & Javorka, 2020; O’Connor et al., 2021; Peterson et al., 2018). Factors contributing to vulnerability, such as increased substance use due to minority stress, likely account for the increased risk of involvement with violence (Shorey et al., 2019). Minority stress, that is stress caused by cultural marginalization due to minoritized identity status, is associated with poorer health outcomes, violence victimization, and violence perpetration (De Schrijver et al., 2022; Shorey et al., 2019). Effective prevention of sexual victimization for LGBTQ+ people therefore must include perpetration prevention and, in turn, research examining perpetration.

Detailed, fine-grained data are needed to understand who perpetrates and why. However, for many valid and practical reasons (e.g., it is simply difficult to know another person’s sexual orientation), we could only locate a few examples in the sexual victimization literature (Martin et al., 2020) that asked participants to report on the gender identity of the perpetrator. Similarly, in sexual violence perpetration research, the gender and sexual identity of the respondent are often measured, supplying a crucial piece of the puzzle. However, the corresponding characteristics of the person they harmed are rarely queried. For example, in one large study of over 13,000 college studies that did inquire about the gender of the perpetrator, cisgender women, and gender diverse students reported cisgender men as the most common perpetrators. However, this study did not inquire about sexual identity (Martin et al., 2020). The current study looks to build upon these important efforts to better understand sexual perpetration and elevate the research to the more developed understanding illustrated in intimate partner violence literature, in which gender and sexual identity patterns are more clearly characterized (Shorey et al., 2019).

Very few studies have asked LGBTQ+ individuals about their own perpetration behavior. Currently available findings are mixed, due to the small number of studies, sometimes small sexual minority subsample sizes within those studies, an even smaller number who report perpetration within those subsamples, and methodological variation. Although our study focuses on only gay men and lesbian women, because these monosexual populations are often grouped with other sexual minority populations, we often use umbrella terms to be consistent with how the data were reported. When possible, we specify which population is of focus. For example, although one study found that heterosexual men were more likely to report sexual violence perpetration than sexual minority men (Walsh et al., 2019), another study found that behaviorally bisexual men (i.e., sexual orientation as classified by behavior, not stated identity) were more likely to report sexual violence perpetration than heterosexual men (Krahé & Berger, 2013). Still, two other studies did not find a significant difference in sexual violence perpetration between heterosexual and sexual minority men (Anderson et al., 2017; Krahé et al., 2021). Trottier et al. (2021) found that heterosexual men and sexual minority women perpetrate sexual violence more frequently than sexual minority men and heterosexual women. Yet, Krahé et al. (2021) found the opposite when classifying participants by behavior rather than sexual orientation—heterosexual women perpetrated more than sexual minority women. However, all five of these studies utilized small sexual minority subsamples, ranging from 24 to 197 participants per subsample before accounting for reported perpetration (see Table 1 for a summary). Likely because of these sometime small numbers, existing studies often combined sexual minority groups in calculating prevalence rates. For example, Trottier et al. (2021) tested prevalence rates using the comparison groups of heterosexual men vs. heterosexual women vs. cisgender sexual minorities vs. transgender/nonbinary individuals. Trottier et al. (2021) are illustrative of subsample size difficulty—although the total sample was 1796 individuals, only 37 lesbian women participated, and of those, only 13 reported perpetration.

**Table 1:** Comparison of perpetration rates and measurement strategies across studies of sexual perpetration for monosexual sexual minority subsamples

**#**	Study and population	Sexual minority sub-sample	*n*	Perpetration rate	Measurement strategy
1	Krahé and Berger (2013)	Gay men*	≈24	7.7%	Modified 2007 SES
	German college students	Lesbian women	≈37	0.0%	
2	Anderson et al. (2017) American college students	Gay/bisexual men	53	20.8%	2007 SES
3	Walsh et al. (2019) American college students	Gay/lesbian/bisexual men and women	34	2.1%	Modified 2007 SES
4	Trottier et al. (2021)	Gay men	63	31.7%	Tactic-first 2007 SES
	Canadian college students	Lesbian women	37	35.1%	
5	Krahé et al. (2021)	Gay/bisexual men*	67	22.4%	SAV-S
	German college students	Lesbian/bisexual women	197	11.2%	
-	The current study	Gay men	164	35.0%	Brief PRSPS-P
	American MTurk workers	Lesbian women	177	20.5%	

*Note*: *as classified by behavior, not identity. *SES*, Sexual Experiences Survey; *SAV-S*, Sexual Aggression and Victimization Scale; *PRSPS-P*, Post-Refusal Sexual Persistence Scales. Although some studies did report data for bisexual individuals, we did not summarize that here given our focus on monosexual individuals only. ≈ These subsample sizes were not reported specifically but estimated from *n*’s and percentage endorsing same-sex contacts only.  Since entering college

Measurement choices were also different in each study. These seemingly small differences in measurement may be especially influential with LGBTQ+ subsamples given that prior research suggests that commonly used measures may not be accurately capturing LGTBQ + people’s experiences (Anderson et al., 2021a, 2021b, 2021c, 2021d; Canan et al., 2019). Another methodological difference is in how sexual orientation was measured—by self-reported sexual interest or preference (i.e., how a person self-identifies) or by sexual experiences or behaviors (i.e., past and present sexual partners). All of this is to say that the identities of those who are perpetrating violence against LGBTQ+ individuals are somewhat unclear; thus further research is necessary, including actually asking LGBTQ+ individuals about their perpetration behavior.

## Perpetration Characteristics

Another under-examined aspect of sexual violence perpetration in general and especially within LGBTQ+ populations is variation in perpetration tactics. Much like other human behaviors, perpetration behavior is heterogeneous, and specific tactics co-vary in meaningful ways based on personal and situational characteristics (Brennan et al., 2019; DeGue et al., 2010). Characterizing this heterogeneity may be important for increasing the efficacy of interventions; currently, available interventions are small in number and often have poor efficacy (DeGue et al., 2014; Wright et al., 2018). Investigation into how tactic use may vary with gender and sexual identity may provide insights into theory and necessary intervention strategies. For example, because of gender stereotypes (e.g., the belief that women are not physically strong), women may be more likely to use verbal tactics rather than physical tactics. Some research suggests that which tactic type is used is related to the perpetrator’s individual characteristics or attitudes; for example, those who are more emotionally sensitive may be more likely to use verbal tactics and manipulation (DeGue et al., 2010). Other research suggests that the tactic used is more related to the desired outcome; tactic use escalates into violence until the desired result is achieved (Testa et al., 2019).

Yet, it is unclear how current findings and theories regarding perpetration may apply to LGBTQ+ individuals. A consistent predictor of sexual perpetration is social power and the attitudes associated with that power, such as hostile masculinity and acceptance of violence (O’Connor et al., 2021). However, due to the marginalization of LGBTQ+ individuals and minority stress, social power and related attitudes as predictors of perpetration may be less relevant or look different. For example, as feminist scholars and theorists have demonstrated, the cultural norms around heterosexuality can foster perpetration, particularly the internalization of gendered norms around sexual agency—the idea that heterosexual men have a right to and should be initiating sex while heterosexual women are gatekeepers whose pleasure is irrelevant (Gavey, 2018; Hakvåg, 2010; Jeffrey, 2024). Thus, LGBTQ+ individuals, particularly those in same-gender relationships, may have the opportunity to develop more egalitarian sexual and relationship dynamics free of coercion. In another example, rates of substance use, including alcohol consumption, are higher among LGBTQ+ individuals compared to non-LGBTQ+ individuals; therefore, they may be more likely to be involved in alcohol-related sexual violence (Coulter et al., 2015; Hughes et al., 2010; Watson et al., 2020). Thus, there are risk factors that suggest the populations considered in this study, gay men and lesbian women, could experience increased risk (i.e., minority stress, substance use) and decreased risk for sexual perpetration (i.e., less gendered sexual norms) suggesting characterizing the nature of perpetration may be beneficial for developing better theory.

## Measurement Issues

As noted by Anderson et al., (2021a, 2021b, 2021c, 2021d) in the aforementioned papers, measurement issues can obscure the nature of risk factors, prevalence rates, and outcomes of sexual violence research, especially when considering sexual violence against marginalized populations. The existing body of research suggests two critical factors for understanding perpetration against LGBTQ+ individuals: (1) the questionnaire utilized can greatly impact reported rates (Anderson et al., 2021a, 2021b, 2021c, 2021d; Anderson et al., 2021a, 2021b, 2021c, 2021d); and (2) some changes to questionnaires may be necessary to accurately detect cases (Canan et al., 2019; Hipp & Cook, 2017). For example, making another person penetrate you with their penis or an object may be more frequently instigated by heterosexual and sexual minority women (Anderson et al., 2020). However, this form of violence is not typically captured by the most common questionnaires (e.g., the Sexual Experiences Survey: SES).

## The Current Study

Given the need to understand perpetration more inclusively, the goal of this exploratory, secondary analysis was to examine sexual violence perpetration rates by gender and sexual identity in a convenience sample of cisgender, monosexual (e.g., heterosexual or gay/lesbian) adults. Because of the nature of secondary data analysis, we could not include bisexual individuals in this study. However, this study still extends prior research (e.g., Trottier et al., 2021) by utilizing a version of the Post-Refusal Sexual Persistence Scale (PRSPS) to assess sexual violence perpetration, a measure specifically designed to capture sexual violence perpetrated by men and women and which does not use gendered or heteronormative language. This is particularly important given that the measurement tools used in four of the five aforementioned, most relevant studies summarized in Table 1 (e.g., the Sexual Experiences Survey) may underestimate sexual violence, particularly forms that fall outside the traditional cisgender-heterosexual male perpetrator/cisgender-heterosexual female victim dyad (Anderson & Delahanty, 2019; Anderson et al., 2018, 2020). We specifically focus on examining sexual orientation as measured by sexual identity—that is how a person labels their orientation—rather than reported sexual behaviors or attractions. Given the different populations in this study (non-college students), different measurement tools used in this study, the sometimes small gay/lesbian subsample sizes in prior research, and the use of secondary data analysis, we did not make any specific hypotheses and consider these analyses exploratory.

## Methods

### Participants

A total of 838 of the original 995 participants were included in this study. Participants were recruited through Amazon’s Mechanical Turk—a crowdsourcing work site through which individuals worldwide can view an online market of studies and brief tasks posted by companies, individual users, and researchers and choose to work or participate in studies for a posted compensation amount. Filtering techniques allowed our study to be only available to American adults. Participants who did not accurately answer one or both attention checks (13.5%) or who reported an identity outside of the study parameters (2.2%) were excluded from the analysis, resulting in a sample of 838 participants. Participants ranged in age from 18 to 78 (*M* = 38.78, *SD* = 13.04). In the full sample, 177 participants identified as lesbian women (21.1%), 164 as gay men (19.6%), 264 as heterosexual women (31.5%), and 233 as heterosexual men (27.8%). Participants chose both their gender and sexual identity from a menu of options and were encouraged to write in their experienced identity if it was not accurately described in the available options. A single predictor variable with four categories was used to reflect these groups (lesbian women, gay men, heterosexual women, heterosexual men). One of our smallest groups was lesbian women, which was still larger than 7 of the eight subsamples in prior studies (see Table 1). The following racial and ethnic identities were represented: White (75.7%), African American or Black (8.6%), Latino or Hispanic (6.3%), Asian (6.2%), Multiracial (1.7%), Middle Eastern (0.7%), and Native American or Alaskan Native (0.5%). Participants reported a range of education levels; 11.6% reported high school as their highest level of completed education, 23.6% reported having completed some college, 14.4% reported an Associate’s degree, 35.6% reported a Bachelor’s degree, 11.6% a Master’s degree, and 2.7% reported having earned a doctorate.

### Procedure

Participants were recruited and compensated via Amazon’s Mechanical Turk between March and September 2018 for a study advertised as aiming to “learn about how people form impressions about people and situations with limited information.” One of the primary goals of the parent study was to examine how stereotypes of bisexuality held by gay men and lesbian women individuals may impact perceptions of bisexual victims of sexual assault. Thus, bisexual individuals were excluded from the parent study due to the focus on monosexuality. First, participants were presented with a vignette depicting a sexual assault, which varied in the sexual identity of the portrayed victim and the tactic used to victimize, and they responded to several questions about the vignette (see Dyar et al., 2021). Then, they completed a series of questionnaires, which are the focus of the current analyses. The vignette and questionnaires were administered through Qualtrics. In order to participate, individuals were required to be 18 years or older, live in the United States, be able to read English, be cisgender men or women, and identify as either lesbian, gay, or heterosexual. The data reported here were collected for an experimental study examining perceptions of bisexual victims^1^ of sexual assault (Dyar et al., 2021). Due to the nature of the data, this dataset is not available publicly but is by request to Drs. Dyar or Anderson. Data collection was conducted in accordance with APA principals under the supervision of the University of Cincinnati IRB.

### Measures

#### Perpetration

A shortened, gender-neutral version of the Post-Refusal Sexual Persistence Scale-Perpetration (PRSPS-P) (Strang et al., 2013: also called the Sexual Strategies Scale) was used to assess participants’ experiences perpetrating sexual violence since age 14. The PRSPS-P assesses a wide variety of tactics that can be used to coercively obtain sexual experiences; the current study utilized six items from the PRSPS-P. These items were chosen using a combination of desire to represent each possible type of tactic (verbal coercion, substance use facilitation, physical force) and prior data (Anderson et al., 2021a, 2021b, 2021c, 2021d) to select the most frequently endorsed tactic for tactic types in which there are multiple items (e.g., verbal coercion). Items prompted participants to identify how many times in the past (0, 1, 2–5, 6–9, 10 +) they had used various strategies “to convince someone to have sex (oral, anal, or vaginal intercourse) or sexual contact (kissing, making out, touching private parts or genitals) with you when they did not want to.” Below this introductory statement was a list of six tactics including: “Telling them lies (e.g., saying “I love you” when you don’t” an example of verbal coercion, “Taking advantage of the fact that they are drunk or high” an example of substance use facilitation, and “Threatening to harm them physically” an example of physical force. The PRSPS-P is correlated with SES scores (r ≈ 0.337 as converted and calculated using data reported in Strang et al., 2013) and has adequate test–retest reliability (Anderson et al., 2021a, 2021b, 2021c, 2021d).

### Data Cleaning and Analytic Plan

Participants completed two attention check items that instructed participants to give a specific response for that item. Additionally, participants were excluded if they did not complete a single item of the PRSPS-P (*n* = 11). Data from PRSPS-P responses were represented in multiple ways (dichotomously, continuously) throughout the data analytic process to account for the complex nature of sexual violence perpetration (Davis et al., 2014). If a participant marked any PRSPS-P item as “1” or greater, they were coded as having a history of perpetration for that item and tactic type. Because we had at least thirty participants from each gender X monosexual sexual orientation identity group *and* who endorsed perpetration, we met CDC recommended standards for small group analyses (Parker et al., 2017) and conducted tactic-based analyses.

Because the presented data came from another study utilizing an experimental manipulation, condition types (i.e., the victim’s sexual identity and the perpetrator’s tactic use in a sexual assault vignette) were included as covariates in all analyses. In the heterosexual and bisexual victim conditions, condition was not significantly associated with perpetration; however, in the lesbian condition, we did find an experimental effect. In the lesbian victim condition, participants reported more perpetration compared to the (*F*(2, 791) = 3.677, *p* = 0.026, η^2^ = 0.009 (small effect), Cohen’s *d* = 0.191) bisexual victim or heterosexual victim conditions (*p* = 0.021). To put in practical terms, 36.8% of those in the lesbian victim condition reported perpetration compared to 33.9% in the heterosexual victim condition and 29.3% in the bisexual victim condition. Other studies have recently been published utilizing such a manipulation while investigating prevalence rates (Oesterle et al., 2023), and given the limited data available on perpetration rates within this population, and small effect size distributed across participants, we believe this data still contributes to the field.

## Results

### Perpetration Prevalence Rates

In the full sample, 201 participants (24.3%) endorsed perpetrating sexual violence. Considering perpetration by gender and sexual identity, 33.0% (*n* = 75) of heterosexual men reported perpetration compared to 35.0% (*n* = 57) of gay men, 20.5% of lesbian women (*n* = 36), and 12.6% of heterosexual women (*n* = 33). There was a significant association between gender and sexual identity group and history of perpetration, *F*(3, 791) = 13.941, *p* < 0.001, η^2^ = 0.050 (medium effect size; see Table 2). The proportion of heterosexual men who reported perpetration significantly exceeded that reported by heterosexual women (*p* < 0.001) and lesbian women (*p* = 0.015), but it was not statistically different from that reported by gay men. Similarly, the proportion of gay men who reported perpetration was significantly higher than the proportion of heterosexual women (*p* < 0.001) and lesbian women (*p* = 0.008). There was not a significant difference between heterosexual and lesbian women (*p* = 0.226).

**Table 2:** Tactic type endorsement among the subsample who reported perpetration

	Heterosexual men (*n* = 75) n, % of column	Heterosexual women (*n* = 33) n, % of column	Gay men (*n* = 57) n, % of column	Lesbian women (*n* = 36) n, % of column	F or Fisher’s exact	η^2^ for F
Tactics summed (0–3)					**F**	
Number of tactic types M(SD)	.52 (.88)	.21 (.63)	.55 (.85)	.29 (.67)	.96	.017
Perpetration tactic					**F**	
Any verbal coercion	39 (52.0%)^a^	15 (45.5%)	18 (31.6%)	11 (30.6%)^a^	2.99* ±	.052
Any alcohol or drugs	26 (34.7%)	12 (36.4%)	24 (42.1%)	6 (16.7%)	1.20	.021
Any threats or use of force	14 (18.7%)	8 (24.2%)	5 (8.8%)	4 (11.1%)	1.68	.030
Combinations of tactics					**Fisher’s**	
Verbal coercion & alcohol	22 (29.3%)	9 (27.3%)	12 (21.1%)	5 (13.9%)	.32–1.00	-
Verbal coercion & force	12 (16.0%)	8 (24.2%)^a^	3 (5.3%)^a^	4 (11.1%)	.02*, .09–.53	-
Alcohol & force	12 (16.0%)	6 (18.2%)	4 (7.0%)	4 (11.1%)	.162–1.00	-
All three	12 (16.0%)	6 (18.2%)	3 (5.3%)	4 (11.1%)	.07–.78	-

Note. Overall prevalence rates of perpetration for the entire sample were: heterosexual men, 33.0%, heterosexual women, 12.6%, gay men 35.0%, lesbian women, 20.5%

± Fisher’s exact = .042, *p* < .05

^*^p < .05, significant differences noted between groups noted ^a^

- analysis not conducted due to limited power for *F* tests

### Perpetration Tactic Patterns

Verbal coercion was endorsed at the highest rate (9.9%) in the full sample, followed by the use of drugs or alcohol (8.1%), and lastly, the threat of force or use of physical force (3.7%). Next, we explored differences in the frequency of using specific tactics among the subsample of those who reported perpetration of some type (*n* = 201). A MANOVA was used to examine differences in perpetrators’ tactic endorsement across gender and sexual identity groups. Among those with perpetration histories, gender, and sexual identity groups significantly differed on their endorsement of verbal coercion (*F*(3, 165) = 2.992, *p* = 0.033, η^2^ = 0.052, medium effect), but not use of alcohol or drugs (*F*(3, 165) = 1.197, *p* = 0.312, η^2^ = 0.021, small effect) or physical force or threats of force (*F*(3, 165) = 1.683, *p* = 0.173, η^2^ = 0.030, small to medium effect). Post hoc Fisher’s exact tests for verbal coercion found a difference between heterosexual men’s (52.0%) and lesbian women’s (30.5%) use; in other words, heterosexual men who perpetrated reported using verbal coercion more often than lesbian women who perpetrated (see Table 2). There were no other statistically significant group differences in tactic use patterns when considering whether verbal coercion, alcohol or drugs, or physical force were reported as having ever been used. Note that the data in this table is from the subsample of those who reported perpetration, and percentages reference the subgroup n, not overall n.

An ANOVA was conducted to compare the number of different tactic types used across gender and sexual identities. Gender and sexual identity group was not significantly associated with number of tactic types endorsed (1–3; physical, verbal, alcohol-related), *F*(3, 165) = 0.955, *p* = 0.416, η^2^ = 0.017. Thus, no group was more or less likely to report using multiple tactics compared to another. An additional analysis was used to compare the endorsement of tactic combinations across gender and sexual identities. Table 2 notes the proportions of participants who reported various tactic-type combinations using Fisher’s exact tests. The only statistical difference was that more heterosexual women (24.2%) than gay men (5.3%) who perpetrated reported using the combination of verbal coercion and physical force in their lifetimes.

## Discussion

Sexual minority individuals and other marginalized groups bear a more significant burden of sexual violence victimization than the average American citizen (Black et al., 2011; Rosay, 2016). To truly prevent sexual violence, we must study and develop interventions to prevent perpetration in addition to providing risk reduction and treatment services for those who are harmed. Yet, LGBTQ+ individuals have been largely excluded from perpetration research (Anderson et al., 2021c), and some of the most commonly used measures of sexual violence may be inappropriate for capturing their unique experiences (Anderson et al., 2021a, 2021b, 2021c, 2021d; Canan et al., 2019). Considering the additional challenges of conducting this research due to the harmful and inaccurate stereotypes that LGBTQ+ individuals are sexual predators (Mishel, 2020), it is even more important to conduct this research using sound methods. This study sought to partially extend Trottier et al. (2021) to date one of the largest studies examining perpetration across multiple sexual minority groups, by using a non-gendered, more inclusive measure of sexual violence to examine potential differences in reported perpetration between cisgender men and women who identified as gay/lesbian or heterosexual. Considering prevalence rates, both heterosexual and gay men reported similar perpetration rates (33–35%), which were significantly higher than those reported by lesbian women (20.5%) and heterosexual women (12.6%). Yet importantly, our data are consistent with prior research suggesting that perpetration by women occurs at greater than negligible rates (Stemple et al., 2017).

Focusing on extending Trottier et al. (2021), our findings were similar for rates of perpetration for heterosexual men (37.9%) and gay men (31.7%), but their estimates for women were much different from our sample. It is possible that because we recruited a larger number of lesbian women (*n* = 177 in this sample vs. 37 in Trottier et al.), our estimated prevalence rate of 20.5% may be more reliable than Trottier’s estimate of 35.1%. However, this would not explain the large differences between our study and Trottier’s study for heterosexual women’s rates of violence perpetration. Trottier et al. (2021) reported prevalence rates among heterosexual women nearly twice as high as ours (Trottier et al.: 23.5% vs. 12.6% in this study). Their sample of heterosexual women was five times larger than ours. The version of the SES used by Trottier et al. (2021) retains many of the same gendered and heteronormative biases of other versions of the Sexual Experiences Survey. At least one study using a version of the Sexual Experiences Survey to assess perpetration in women reported high false-positive rates (Buday & Peterson, 2015), suggesting that our findings may be a result of measurement differences. Alternatively, these differences in prevalence rates may reflect population differences between MTurk adults (this study) and Canadian college women in Trottier’s study. Thus, we extend the literature by replicating Trottier et al.’s findings for men and documenting different rates of perpetration for women with a robust methodology.

Considering a focus on tactics, the only group difference we found in overall tactic use by type was that heterosexual men reported more use of verbal coercion than lesbian women; we found no group differences for the perpetration tactics of substance use (8.1% prevalence in the sample) and physical force (3.7% in the sample). This is in contrast to Krahe & Berger (2013), who found group differences in substance use tactics but who defined sexual orientation behaviorally rather than by identity. Thus, it is unclear by what aspect of sexual orientation, one’s self-reported sexual preference or reported experiences, tactic use varies. In general, prior studies have not examined covariation of tactics; we found that heterosexual women were more likely to report using verbal coercion and force *in combination* than gay men. This suggests that a focus on tactics may highlight important group differences that could be targeted in intervention.

In summary, our findings suggest that gender may be more closely related to the occurrence of sexual violence perpetration than sexual identity, given the medium effect sizes found for differences in the occurrence of perpetration behaviors. However, it is notable that few heterosexual women reported perpetration (*n* = 33), limiting analyses. Although there were few differences within gender, future research should investigate the potentially different mechanisms underlying these behaviors for these groups, given the nascent nature of this research area. In other words, while these behaviors appear to be occurring at the same rate within gender groups, there may be different causes related to sexual identity. Gay men may feel less pressure to conform to certain male norms that facilitate violence and therefore could decrease risk for perpetration (Baugher & Gazmararian, 2015); yet, gay men are more likely to use substances and experience minority stress, both of which are associated with violence perpetration (Shorey et al., 2019; Testa et al., 2019). Finally, although women were less likely to report perpetration than men in this sample, the number of women who reported perpetration was not small, and women’s perpetration across identity groups is worthy of further research. The findings on women’s perpetration rates are especially notable considering that we used a measure of sexual perpetration behavior that was specifically developed and validated with women, unlike other extant measures used in prior research.

Notably, once we examined behaviors within those who reported perpetration, small sexual identity-related differences did emerge. Notably, two tactic differences were identified in the eight analyses conducted suggesting that overall, sexual identity is a small factor. We found that heterosexual men who perpetrated used verbal coercion more than lesbian women, and heterosexual women who perpetrated reported the combination of verbal coercion and force more often than gay men who perpetrated. Thus, while gender seems to be the most influential factor in whether perpetration occurs or not, sexual identity may influence the tactics used. We encourage future research to use theories such as coercive heterosexuality (Jeffrey, 2024), disempowerment theory (McKenry et al., 2006; Milletich et al., 2014), and the Integrated Theory of Sexual Minority Alcohol-Related Violence Perpetration (Shorey et al., 2019) to better understand sexual violence perpetration among gay and lesbian individuals. These theories conceptualize how and why sexual minority individuals, particularly women, are more likely to experience risk factors for violence perpetration. Lesbian women may feel additional marginalization due to their dually minoritized identities as women and as sexual minorities. These models integrate risk factors that cut across genders and sexual identities, such as alcohol use and minority-specific risk factors, such as minority stress.

### Clinical, Research, and Policy Implications

The rates of perpetration across sexual identities suggest that practitioners working should attend to this risk behavior by screening patients for a history of perpetration and working to ameliorate risk factors for violence, such as reducing substance use. We recommend that any clinical work with LGBTQ+ individuals utilize an affirming psychotherapy model (Burton et al., 2017). The differences in tactic use among those with perpetration histories suggest that the etiology of perpetration may differ across groups. In other words, although the rate of perpetration may be the same, the context in which it occurs may differ. More research on specific tactics, which may have different etiologies (DeGue et al., 2010), is needed. We also suggest further research on the measurement of violence across cultural groups; although we used a measure that we hope decreases heteronormative bias, more research is needed to confirm this and related methods. This study suggests that policies that support greater research and clinical resources to prevent perpetration, especially for men, are needed. Finally, policies that address sexual violence, such as Title IX, should be more inclusive of LGBTQ+ individuals and policies specific to LGBTQ+ needs should be considered to adequately address sexual violence against LGBTQ+ individuals.

### Limitations

Although our samples of gay men and lesbian women were much larger than in prior research and similar to the heterosexual subsamples included in our study, the sample sizes were still small for the purposes of determining prevalence rates, as demonstrated in the small effect sizes in follow-up analyses. This sample size also limited intersectional focused analyses that might illuminate how sexual identity interacts with other salient identities or life experiences. Furthermore, due to the use of a convenience secondary sample, we were not able to include a plurisexual (bisexual +) comparison group; this is especially notable as plurisexual individuals are the largest sexual minority subgroup and they are at particularly high risk of sexual violence victimization (Chen et al., 2020). We did not ask participants to report on the gender or sexual identity of those that they harmed; thus, our data can speak only to perpetration rates, not the context or targets of perpetration. Our use of an abbreviated measure of perpetration potentially truncated reported prevalence rates although rates were still consistent with other research in spite of our measure being much shorter. Finally, because this data represents a secondary analysis, reports of perpetration were impacted by the experimental manipulation such that those who were exposed to the lesbian victim vignette were more likely to report perpetration.

## Conclusion

There are gender and sexual identity differences in the rates and characteristics of sexual violence perpetration. Heterosexual and gay men (33.0, 35.0%) reported more sexual violence perpetration than lesbian and heterosexual women (20.5%, 12.6%). Considering the tactics used by those who perpetrated, heterosexual men who perpetrated were more likely to use verbal coercion tactics than lesbian women who perpetrated. Heterosexual women who perpetrated were more likely to jointly use verbal coercion and force than gay men who perpetrated. In spite of the discomfort to study potentially controversial topics, to truly prevent sexual violence, we must study perpetration within and outside the LGBTQ+ community to learn how to change this harmful behavior. Further research should examine the mechanisms that facilitate perpetration behavior across gender and sexual identity groups (Turchik et al., 2015).

## Footnotes

1 We use the term victim to generally describe those who are targeted by perpetrators for violence. However, we affirm people’s self-agency in deciding whether to label themselves as a victim, survivor, or something else (including not using a label).
